# Concordance within parent couples’ perception of parental stress symptoms among parents to 1-18-year-olds with physical or mental health problems

**DOI:** 10.1371/journal.pone.0244212

**Published:** 2020-12-18

**Authors:** Signe Boe Rayce, Maiken Pontoppidan, Tine Nielsen

**Affiliations:** 1 Department of Health, VIVE, The Danish Centre for Social Science Research, Copenhagen, Denmark; 2 Department of Psychology, University of Copenhagen, Copenhagen, Denmark; University of Warsaw, POLAND

## Abstract

Parents of children with physical or mental health problems are at higher risk for experiencing parental stress. However, mothers and fathers may experience parental stress differently. The aim was to examine whether mothers and fathers of children with physical and/or mental health problems are equally inclined within the couples to experience different aspects of parental stress when considering child and parent couple characteristics. Single aspects of Parental stress were assessed with nine items from the Parental Stress Scale in 197 parent couples of children aged 1–18 years with physical and/or mental health problems. Agreement within parent couples for each item was tested using two tests of marginal homogeneity for dependent data: a nominal G^2^-test and an ordinal γ-test. Analyses were conditioned on child gender, child age, couple educational level, and overall parental stress. For seven aspects of parental stress, differences in agreement within the couples were found with at least one of the conditioning variables. For five aspects (item 3, 4, 9, 10, 13) addressing specific personal experience of daily stressors related to having children and feeling inadequate as a parent, the differences were systematic. Mothers were more inclined to experience these aspect of parental stress than fathers, specially mothers of boys, a younger child, in couples with an education above high school or with a higher stress level. Agreement was found for two aspects (item 14 and 16) of parental stress. This study suggests that mothers’ and fathers’ experience of most aspects of parental stress vary within the couples. Knowledge on systematic difference between parents’ experience of parental stress may inform future interventions. For aspects where mothers generally experience the highest degree of stress, fathers may be involved as support. Future studies may explore the role of diagnoses, coping strategies and examine concordance in parental stress symptoms in other subgroups.

## Introduction

Becoming a parent is a rewarding experience for most parents, but also stressful, because of the day-to-day hassles of parenting. Parental stress can be defined as “a set of processes that lead to aversive psychological and physiological reactions arising from attempts to adapt to the demands of parenthood” [1]. There is an elevated risk of parental stress for families experiencing e.g. poverty, mental health problems, and adverse childhood experiences [2–7]. Families with children with developmental problems such as Attention Deficit Hyperactivity Disorder (ADHD) or Autism Spectrum Disorder (ASD) are especially at risk and report higher levels of parental stress than both families of typically developing children and parents of children with other disabilities such as Downs syndrome and Cerebral Palsy [8–18]. This is likely due to the increased caregiving demands that are associated with parenting children with these conditions [10]. For parents of children with ASD research suggest that the following three factors contribute to the elevated parental stress levels: 1) child characteristics (particularly behavior problems), 2) lack of professional support and access to services, and 3) a lack of understanding for problems experienced by the child and the family [19]. Research shows that highly stressed parents tend to exert harsh or ineffective parenting strategies [20] and be less responsive towards the child [21]. Such parenting strategies may lead to disruptive child behavior, which again increases parental stress [22]. Appropriate interventions offered to families with children with developmental disabilities can reduce the level of parental stress [23, 24]. It is therefore important to assess parental stress and support parents who experience high levels of parental stress.

Most research on parental stress is conducted on mothers only, but research including fathers is increasing. Most families consist of a father and a mother and it is therefore important to include data on parental stress experienced by the father. The existing evidence on parental stress differences between fathers and mothers is equivocal. While some studies find differences in the absolute levels of parenting stress between mothers and fathers [13, 19, 25–34], other studies do not find any difference [34–40]. However, when differences are found, mothers typically report higher parental stress than fathers [13, 25, 26, 34–40]. Examining parental stress is complex and although many studies have been conducted recently, Crnic and Ross state that *“the field seems no closer to resolving whether mother-father differences exist in the experience of parental stress than it was a decade ago”* [3, p. 278].

Focusing on parents of children with developmental disorders (such as ADHD and ASD) and behavior problems, a relatively limited number of studies have examined whether mothers and fathers differ in their level and experience of parental stress [13, 25, 26, 34, 41–47]. The studies are conducted in different settings including children with e.g. ASD, autism, oppositional defiant disorder (ODD), and behavior problems and in a wide range of counties including USA, UK, Pakistan, Poland, Denmark, and Ireland. These studies of differences in mothers and fathers parental stress levels consistently find higher levels of parental stress in mothers compared to fathers [13, 25, 26, 34, 41–47]. This is supported by a meta-analysis of parental stress in families of children with ADHD, where mothers experience higher levels of parental stress than fathers do across the seven included studies [48]. However, no information is provided to identify the seven studies included in the meta-analysis and it may include the results of Baker [43]. Four of the studies we identified [49–52], found no significant differences between mothers’ and fathers’ stress level. However, these are, based on relatively small samples and the results may therefore be due to lack of power.

In correlation analyses some studies find that overall parental stress decreases with increased child age [53–55], whereas recent studies do not find differences in parental stress relative to child age [17, 18]. With regard to child gender some studies indicate that mothers of girls may experience less parental stress than mothers of boys [18, 54, 56]. Characteristics such as child age and gender are shared between the parent couple and it is possible that this leads to similar levels of parental stress within the couple. Parental stress is also influenced by the general stress level generated outside the family context such as job stress, and interpersonal relationship stress [3]. For example, studies suggest that mothers with low or high education report a higher degree of parental stress compared to mothers with an intermediate educational background [30, 57–59]. If parents experience very different stress levels in their daily life outside the family it is possible that these individual parental characteristics and experiences contribute to differences in parental stress *within the couple*.

The existing studies of discrepancies between mothers’ and fathers’ experience of parental stress are based on independent scores (i.e. mothers and fathers from separate families) and do not address to which degree difference in parental stress exist *within the couple*. Furthermore, we do not know from previous research whether mothers and fathers differ in their experience of specific symptoms or aspects of parental stress (i.e. at the item level). It is possible that there is concordance between mothers and fathers on some specific parental stress symptoms and discordance on others. Mothers and fathers may be with their children in different stressful situations during the day (e.g. picking up from daycare, household chores, putting to bed) which may cause differences in their individual parental stress symptoms [3, 20, 60, 61]. It is also possible that one parent in a couple finds it more stressful to have e.g. younger children than the other parent does. Relative concordance within the couple–how the two parents agree or disagree on specific symptoms of parental stress–is an important aspect of parental stress since knowledge about this could direct intervention strategies at the areas of discordance and thereby potentially lower the level of experienced parental stress within the couple.

Some researchers suggest, that mothers and fathers experience parenting stress differently [62]. They find that while high levels of parenting stress in mothers is related to their parental and spousal roles, elevated parental levels for fathers is related to social isolation [62]. Parents cope with stress in different ways e.g. through individual coping strategies and support from others. Partners in a couple will also resort to dyadic coping strategies which refers to “partners’ coping responses to each other’s stress resulting from circumstances outside relationship” [63, 64]. If the parents are able to apply positive dyadic coping strategies both parents benefit from it and become better at e.g. handling stress [63, 65], while negative coping strategies may result in e.g. blaming, distancing, insincere support [63, 66, 67]. Differences within the couple in both individual and dyadic coping strategies may therefore also contribute to differences in parental stress levels.

To our knowledge concordance and discordance in aspects of parental stress among mothers and fathers has not yet been examined using methods that take into account their relatedness as part of a couple. Therefore, the aim of this study is to explore this new and potentially important field: 1) whether mothers and fathers *in couples* are equally inclined to experience different symptoms of parental stress, and 2) whether any concordance or discordance in the symptom agreement of mothers and fathers *in couples* are homogeneous depending on a) child gender, b) child age, c) parent couple educational level and d) parent couple stress level. We explore this by applying analysis of marginal homogeneity of mothers’ and fathers’ paired responses to the items of the stress (sub)scale of the Danish Parental Stress Scale [59] from a sample of parent couples likely to experience a significant degree of parental stress: parents of children aged 1–18 with physical or mental problems.

## Methods and material

### Sample

The study sample comprises 197 parent couples (i.e. 394 parents) of children aged 1–18 years with physical and/or mental problems, representing a group of parents in risk of experiencing parental stress. Data comes from three separate intervention studies and comprise baseline data only, i.e. data was collected before the intervention started (and before randomization if there was one): (1) Caring in Chaos: 107 parent couples of children aged 3–9 years with ADHD symptoms who participated in the intervention study Caring in Chaos [68] in 2013 or 2014. Caring in Chaos is a behavioral parent training intervention provided by the ADHD patient association and delivered by voluntary trainers. The children were not pre-screened nor excluded based on having a formal ADHD diagnosis. However, all participating parents signed up for the intervention study through the ADHD patient association, and indicated that their child was showing ADHD symptoms. (2) ‘Creating a family-centered childhood disability service’: 82 parent couples of children aged 0–18 years with severe physical and mental health problems who participated in the intervention study ‘Creating a family-centered childhood disability service’ which was conducted in four Danish municipalities in Central Denmark Region by The Method Center—Center for Innovation and Methodology in 2016. The intervention focuses on family centered case management and individual support. Data on specific diagnoses were not available for the present study. The intervention targeted parents of children with severe physical or mental health problems who were referred to treatment within the child services system because the children required extensive daily care and attention from parents and professionals. Disabilities include e.g. ASD, ADHD, cerebral palsy, muscular dystrophy and comorbidity with autism and ADHD being most prevalent in the final sample. (3) PMTO: Eight parent couples of children aged 5–12 years with behavior problems who participated in the Parent Management Training Oregon (PMTO) program conducted by the Child Center of Aarhus Municipalities, a large Danish municipality, in 2018. PMTO is a group-based parent training intervention based on social learning theory and delivered by trained therapists. Written informed consent was obtained from all individual participants included in the primary studies. None of the studies providing data for this study require ethical approval according to Danish law. The Caring in Chaos intervention was approved by the institutional research committee at SFI–the Danish National Centre for Social Research.

Table 1 shows the characteristics of the sample. The sample comprised parent couples of mainly boys (73.6%) and children with a mean age of 8.41 (SD = 3.89). Compared to fathers, the level of education was significantly higher among mothers (p<0.001) with more than half of the mothers (55.1%) having a medium- or long-cycle higher education. The corresponding proportion among fathers was 34.5%. 10.1% of the mothers and 13.3% of the fathers had a high school education or less. Data on parental age or migration status was not available for the present study.

**Table 1:** Sample characteristics.

	Mean	SD	Range
Child age (years) (n = 197)	8.41	3.89	1–18
	**Frequency**	**%**	
*Child gender (n = 197)*			
Boys	145	73.6	
Girls	52	26.4	
*Maternal education (n = 189)*			
High school or less	22	10.1	
Vocational education	53	28.0	
Short-cycle higher education	10	5.3	
Medium-cycle higher education	85	45.0	
Long-cycle higher education	19	10.1	
Other education	3	1.6	
*Paternal education (n = 180)*			
High school or less	24	13.3	
Vocational education	66	36.7	
Short-cycle higher education	18	10.0	
Medium-cycle higher education	34	18.9	
Long-cycle higher education	28	15.6	
Other education	10	5.6	

### The Parental Stress Scale

The Parental Stress Scale (PSS) [35] measures the stress associated with raising children’. The scale focuses on the individual’s perception of parental stress and consists of 18 items related to both positive (10 items) and negative aspects of parenting (8 items). Using exploratory factor analysis, Berry and Jones [35] originally proposed four subscales. One later study has also suggested four subscales although with different distributions of items to the subscales [30]. However, most validation studies find that the PSS consists of two unidimensional scales: parental stress (PS) and lack of parental satisfaction (LPS) [32, 34, 36, 59], which are consistent with Berry and Jones’ original division as this was in fact a further subdivision of the parental stress and parental satisfaction items. Common for all these studies was that at least one item (item 2: *There is little or nothing I wouldn’t do for my child(ren) if it was necessary*) was eliminated. Using the thorough approach of graphical loglinear Rasch modeling [69–71], two recent studies validated the Danish version of PSS among parents of 0-1-year-olds [59] and 2-18-year-olds [34]. Both studies found support for the PS and LPS subscales after dichotomizing the response categories and eliminating an item in each subscale (item 2 and 11). The targeting of the PS subscale to the study populations was excellent in both studies, while the targeting of the LPS subscale was poor. Consequently, in this study we use only the PS subscale items, excluding the problematic item 11 in accordance with the findings of Pontoppidan et al. [59] and Nielsen et al. [34]. In a sample of parents of 2–18 year-olds with behavior problems, Nielsen et al. (2020) reported reliabilities of 0.71 and 0.72 depending on educational background of the parents [34].

The nine items of the PS subscale used in the current study are presented in Table 2. Each item presents a statement about stress related to parenting along with a 5-point response scale (1 = strongly disagree, 2 = disagree, 3 = undecided, 4 = agree, 5 = strongly agree).

**Table 2:** The parental stress (PS) subscale of the PSS.

Item	Parental Stress subscale (PS)
3	Caring for my child(ren) sometimes takes more time and energy than I have to give
4	I sometimes worry whether I am doing enough for my child(ren)
9	The major source of stress in my life is my child(ren)
10	Having child(ren) leaves little time and flexibility in my life
12	It is difficult to balance different responsibilities because of my child(ren)
13	The behavior of my child(ren) is often embarrassing or stressful to me
14	If I had it to do over again, I might decide not to have child(ren)
15	I feel overwhelmed by the responsibility of being a parent
16	Having child(ren) has meant having too few choices and too little control over my life

### Covariates

Four covariates which may affect the experience of parenting and parental stress differently among mothers and fathers are included in the analyses: 1) child gender, 2) child age, 3) parent couple educational level and 4) parent couple stress level.

Child age was divided into three age categories: 1–6 years old, 7–10 years old, and 11–18 years old, as these were the age categories used in the Danish PSS validity study by Nielsen et al [34]. Parent couple educational level was operationalized by combining information on the educational level of each of the parents and subsequently divided into the following three groups: 1) two parents with high school degree or less, 2) One parent with high school or less and one parent with vocational or tertiary education and 3) Two parents with vocational or tertiary education. Only 17 parent couples comprised two parents with high school or less, limiting the statistical power in this stratum of parents. Consequently, only parent couples from the remaining two categories were included in the conditional analyses.

Parental stress level was divided into higher and lower stress. The cut point was the median cut sum score (median = 6) of the PS subscale with dichotomized items (strongly agree, agree and neither agree or disagree coded 1 vs. disagree and strongly disagree coded 0) as in Pontoppidan et al [59] and Nielsen et al [34]. Consequently, a score of six or above in the PS scale was defined as ‘high’ while ‘low’ was defined as a score below six. Parent couple stress level was subsequently categorized as follows: 1) both parents with a low PS score, 2) one parent with a low and one with a high PS score, and 3) both parents with a high PS score.

### Analysis of marginal homogeneity

To assess differences between the responses to individual parental stress items of the mothers and fathers in parent couples, we use tests of marginal homogeneity (MH) for paired/dependent data [72]. Analysis of marginal homogeneity of categorical variables is similar to paired t-tests for continuous variables that take the correlation between the variables into account. A MH analysis estimate the log-linear interaction parameters of the two-way table and compares the observed marginal distributions of the table to the expected marginal distribution under the assumption that the log-linear interaction parameters are the same as in the observed table.

We employ two tests of MH both as implemented in the software package DIGRAM [73]: a likelihood ratio test G^2^ treating the variables as nominal and Goodman & Kruskal’s gamma taking into account the ordinality of the variables. The test by the gamma coefficient is equivalent to the Mann-Whitney test suggested by Agresti [74] and is in accordance with the suggestion by Bergsma, Croon & Hagenaars [75]. In both cases, the distributions of the test statistics and the null-hypothesis that DIGRAM uses to calculate p-values takes the association between the variables into account.

In addition to overall (i.e. crude) analyses of MH, we also tested for conditional marginal homogeneity (CMH) by stratified MH analyses in three-way tables where tests of marginal homogeneity are calculated in different strata and where the test statistics are also summarized as *partial* test statistics for tests of conditional marginal homogeneity.

The conditional analyses were stratified by one covariate at a time (child gender, child age, parent couple educational level, and parent couple stress level, respectively), as the number of parent couples did not allow for simultaneous stratification across the four covariates of the study. We report results both for the single strata in each covariate (i.e. the single conditional results) and across the strata for each covariate (i.e. the partial results taking all strata in the covariate into account). The crude MH results are not considered in the final results, but they are reported for completeness (in the S1 Table).

In accordance with Cox et al.’s [76] recommendation, we distinguished between weak to moderate evidence (*p*-values larger than .01) and stronger evidence (*p*-values smaller than .01) against MH, rather than using p-values in a deterministic manner. If both the G^2^ and γ-test show strong evidence against MH, we consider the γ-test the stronger result, as this takes into account the ordinal nature of the items. Furthermore, the γ-test allows for interpretation on whether mothers or fathers tend to agree more with the statement. The Benjamini-Hochberg procedure was used to assess the false discovery rate (FDR) due to multiple testing [77].

## Results

### Conditional and partial MH analyses

Tables 3–6 show the results of both the conditional and partial MH analyses. Positive γ-coefficients mean that mothers are more prone than fathers to agree with the particular item statement while negative γ-coefficient means that the fathers are more prone to agree. When evidence against MH (i.e. mothers and fathers differ in their agreement on the statement) is provided by the G^2^ only, no information on the character of the marginal differences can be gained from the test result. In such cases additional information can be obtained from the marginal tables, only (S2 and S3 Tables).

**Table 3:** Test for marginal homogeneity between mothers’ and fathers’ responses conditioned child gender.

	CONDITIONAL MH										PARTIAL MH				
	*Boys (n = 145)*					*Girls (n = 52)*									
	G^2-^test			γ-test		G^2-^test			γ-test		G^2-^test			γ-test	
Item	G^2^	Df	P	γ_m-f_	p	G^2^	df	P	γ_m-f_	p	G^2^	df	p	γ_m-f_	P
3	22.9	4	.001	**.30**	**< .001**	9.4	4	.051	.28	.014	32.4	8	< .001	.30	< .001
4	29.4	4	< .001	**.36**	**< .001**	6.4	4	.170	.14	.167	35.8	8	< .001	.34	< .001
9	18.6	4	.001	**.28**	**< .001**	16.3	4	.003	.20	.046	35.0	8	< .001	.27	< .001
10	6.3	4	.180	.012	.044	**41.2**	**4**	**< .001**	.11	.161	47.5	8	< .001	.11	.030
12	4.0	4	.410	-.07	.155	.9	4	.920	.05	.334	4.9	8	.768	-.05	.190
13	16.4	4	.002	.17	**.002**	**23.8**	**4**	**< .001**	.14	.103	40.2	4	< .001	.17	.001
14	2.3	4	.681	-.05	.295	4.4	3	.221	-.08	.264	6.7	7	.460	-.05	.255
15	1.7	4	.794	-.01	.460	7.6	4	.106	.20	.037	9.3	8	.316	.02	.412
16	3.2	4	.532	-.12	.053	1.5	4	.831	.04	.334	4.6	8	.796	-.10	.065

Notes. Benjamini-Hochberg rejects if p<0.0167 for FDR = 5% and if p<0.0031 for FDR < 1%. Strong evidence against MH in the single stratum after taking FDR into account is marked in bold, however, in cases where both the G^2-^test and the γ-test provide strong evidence, only the γ-test results are bolded.

**Table 4:** Test for marginal homogeneity between mothers’ and fathers’ responses conditioned by child age.

	CONDITIONAL MH															PARTIAL MH				
	*Child age 1–6 years (n = 69)*					*Child age 7–10 years (n = 84)*					*Child age 11–18 years (n = 44)*									
	G^2-^test			γ-test		G^2-^test			γ-test		G^2^			γ-test		G^2-^test			γ-test	
Items	G^2^	df	P	γ_m-f_	P	G^2^	df	p	γ_m-f_	p	G^2^	df	P	γ_m-f_	p	G^2^	df	p	γ_m-f_	p
3	12.0	4	.017	**.36**	**.001**	17.1	4	.002	.27	.005	8.4	4	.079	.25	.022	37.5	12	< .001	.30	< .001
4	65.4	4	< .001	.33	.005	23.5	4	< .001	.33	.002	6.5	4	.167	.22	.049	95.3	12	< .001	.31	< .001
9	17.3	4	.002	**.29**	**.001**	23.1	4	< .001	**.31**	**< .001**	5.4	4	.248	.14	.107	45.9	12	< .001	.28	< .001
10	7.3	4	.121	.08	.216	1.8	4	.767	.10	.149	14.9	4	.005	.19	.042	24.0	12	.020	.10	.043
12	1.4	4	.847	-.02	.434	.5	4	.974	-.03	.362	7.8	4	.100	-.04	.339	9.6	12	.647	-.03	.321
13	7.8	4	.098	.23	.012	7.8	4	.099	.17	.018	7.8	4	.111	.06	.277	23.1	12	.027	.18	.001
14	3.3	4	.514	-.14	.161	3.6	4	.460	-.10	.248	3.2	4	.531	.08	.195	10.0	12	.612	-.08	.185
15	3.5	4	.483	-.02	.440	5.2	4	.271	.03	.400	9.0	4	.062	.21	.045	17.6	12	.128	.04	.300
16	7.0	4	.135	-.06	.296	6.4	4	.169	-.17	.037	1.1	4	.898	.05	.295	14.5	12	.268	-.10	.063

Notes. Benjamini-Hochberg rejects if p<0.0120 for FDR = 5% and if p<0.0011 for FDR < 1%. Strong evidence against MH in the single stratum after taking FDR into account is marked in bold, however, in cases where both the G^2-^test and the γ-test provide strong evidence, only the γ-test results are bolded.

**Table 5:** Test for marginal homogeneity between mothers’ and fathers’ responses across parent couple educational status.

	CONDITIONAL MH										PARTIAL MH				
	One parent above high school level *(n = 47)*					Both parent above high school level *(n = 133)*									
	G^2-^test			γ-test		G^2-^test			γ-test		G^2-^test			γ-test	
Item	G^2^	df	P	γ_m-f_	P	G^2^	df	p	γ_m-f_	P	G^2^	df	p	γ_m-f_	P
3	13.3	4	.010	.27	.020	21.2	4	< .001	**.31**	**< .001**	34.5	8	< .001	0.30	< .001
4	**48.5**	**4**	**< .001**	.23	.043	29.0	4	< .001	**.34**	**< .001**	77.5	8	< .001	0.33	< .001
9	7.0	4	.138	.16	.102	15.4	4	.004	**.26**	**< .001**	22.3	8	.004	0.25	< .001
10	4.0	4	.410	.12	.114	6.3	4	.176	.10	.087	10.3	8	.245	0.10	.060
12	0.5	4	.976	.02	.422	2.1	4	.711	-.04	.286	2.6	8	.956	-0.03	.304
13	5.7	4	.226	.10	.153	10.3	4	.036	.14	.014	15.9	8	.043	0.14	.010
14	3.2	4	.528	-.16	.641	0.5	4	.972	.01	.476	3.7	8	.883	-.01	.461
15	2.3	4	.680	.04	.394	2.6	4	.631	.07	.172	4.9	8	.770	0.07	.163
16	3.0	4	.565	-.07	.297	1.8	4	.763	-.06	.211	4.8	8	.778	-.06	.185

Notes. Benjamini-Hochberg rejects if p<0.0111 for FDR = 5% and if p < 0.0017 for FDR < 1%. Strong evidence against MH in the single stratum after taking FDR into account is marked in bold, however, in cases where both the G^2-^test and the γ-test provide strong evidence, only the γ-test results are bolded.

**Table 6:** Test for marginal homogeneity between mothers’ and fathers’ responses across parent couple stress level.

	CONDITIONAL MH															PARTIAL MH				
	*Both parents low PS (n = 58)*					*One parent low one high PS (n = 76)*					*Both parents high PS (n = 63)*									
	G^2-^test			γ-test		G^2-^test			γ-test		G^2-^test			γ-test		G^2-^test			γ-test	
Item	G^2^	df	P	γ_m-f_	P	G^2^	df	p	γ_m-f_	p	G^2^	Df	p	γ_m-f_	P	G^2^	Df	p	γ_m-f_	P
3	11.1	4	.025	.24	.027	15.8	4	.003	.31	.003	16.3	4	.003	**.42**	**< .001**	43.2	12	< .001	0.32	< .001
4	8.3	4	.080	.30	.009	**21.7**	**4**	**< .001**	.27	.012	14.8	4	.005	.39	.003	44.9	12	< .001	0.31	< .001
9	19.3	4	.001	**.37**	**< .001**	15.0	4	.005	.29	.006	9.5	4	.049	.27	.006	43.8	12	< .001	0.31	< .001
10	10.0	4	.041	.25	.010	1.9	4	.760	.03	.386	3.0	3	.385	.15	.112	14.9	11	.188	0.12	.035
12	**81.4**	**4**	**< .001**	.01	.459	4.4	4	.422	.01	.457	**94.0**	**4**	**< .001**	-.01	.463	179.9	12	< .001	0.01	.457
13	4.8	4	.305	.08	.246	9.6	4	.048	.15	.101	35.0	4	< .001	**.38**	**< .001**	49.4	12	< .001	0.20	.001
14	1.7	2	.426	-.32	.101	3.7	4	.449	-.07	.323	2,6	4	.628	-.03	.383	8.0	10	.629	-.08	.170
15	**24.6**	**4**	**< .001**	.08	.240	3.4	4	.497	.10	.230	3.1	4	.535	-.03	.386	31.1	12	.002	0.05	.243
16	1.9	3	.586	.12	.195	3.4	3	.339	-.16	.123	3.4	4	.497	-.12	.147	8.7	10	.563	-.09	.136

Notes. Benjamini-Hochberg rejects if p<0.0185 for FDR = 5% and if p<0.0017 for FDR < 1%. Strong evidence against MH in the single stratum after taking FDR into account is marked in bold, however, in cases where both the G^2-^test and the γ-test provide strong evidence, only the γ-test results are bolded.

MH across mother and father ratings irrespective of child gender, child age group, family education level, and parental stress levels was only accepted for two of the PS items (item 14: *If I had it to do over again*, *I might decide not to have child(ren)*, and item 16: *Having child(ren) has meant having too few choices and too little control over my life*). For these two items the respective partial MH results, therefore, reflect most precisely the agreement between mothers and fathers in the parent couples, dependent on the covariate one wants to take into account (Tables 3–6). For the remaining items, strong evidence against MH was found in relation to at least one covariate.

#### Child gender

Considering child gender only, MH across mother and father ratings were accepted for four parental stress symptoms (item 12, 14, 15, and 16) (Table 3). This indicates that irrespective the gender of the child, mothers and fathers in couples may be equally inclined to agree that it is difficult to balance responsibilities because of the child(ren), that they might not decide to have children if they had it to do over again, that they feel overwhelmed by the responsibility of parenting, and that having children has meant having too few choices and too little control over their life. In the boys’ stratum MH was likewise accepted for item 10 (*Having child(ren) leaves little time and flexibility in my life*) while strong evidence against MH was found for the remaining items. Mothers of boys were systematically more inclined to agree than the fathers in the couples with item 3 (*Caring for my child(ren) sometimes takes more time and energy than I have to give*), item 4 (*I sometimes worry whether I am doing enough for my child(ren))*, item 9 *(The major source of stress in my life is my children)* and item 13 (*The behavior of my child(ren) is often embarrassing or stressful to me*). The marginal correlations were relatively strong for item 3, 4, and 9 ranging between γ = 0.28–0.36 (p<0.001), while the marginal correlation was weaker for item 13 (γ = 0.17, p = 0.002). Among parents of girls, differences between fathers and mothers were found for item 9, 10 (*Having child(ren) leaves little time and flexibility in my life*), and 13 only.

#### Child age

Table 4 shows the analyses conditioned on child age. When considering child age only, MH across mother and father ratings were accepted for item 12, 14, 15, and 16, and furthermore for item 13. Strong evidence against MH was mainly found in the strata of parents of 1-6-year-olds and parents of 7-10-year-olds. Within these two age groups, mothers are systematically more prone to agree, than were the fathers in the couples, that caring for their children takes more time and energy than they have (item 3), that they sometimes worry whether they are doing enough for their child (item 4), and that their child is the major source of stress in their life (item 9). For all three items the marginal correlations were relative strong (range γ = 0.27–0.36, (p<0.005)). Among parents with a child aged 11–18 years strong evidence against MH was found in relation to item 10 only and of an unsystematic character. The non-systematic differences appear to be that more fathers tend to disagree that children leave little time and flexibility in their life, while more mothers tend to agree totally (S2 Table).

#### Couple education

When only couple educational status is taken into account, MH across mother and father responses was accepted for six out of nine items: item 10, 12, 13, 14, 15, and 16 (Table 5). Systematic differences between mother and father ratings were only found in couples where both parents had an educational level above high school. Again, mothers were more inclined to agree with the statements of item 3, 4, and 9 compared to the fathers in the couples (γ ranging between 0.26 and 0.34, (p<0.001)). In the stratum of parents where only one parent had an education above high school there was also strong evidence against MH, but only for item 4. In this group the differences appear to be that mothers tend to totally agree that they sometimes worry whether they are doing enough for their child(ren), while the fathers are undecided (S3 Table).

#### Couple level of parental stress

Table 6 shows the results of the analyses conditioned on couple stress level only. MH across mother and father ratings were only accepted for item 14 and 16. We found strong evidence that mothers are systematically more prone to agree with items 3, 4, 9 and 13 when both parents have a high overall stress level than were the fathers in the couples. In the stratum where both parent had low levels of overall stress, moderate to strong evidence against MH was likewise found for item 4 and 9, and in addition for item 10. In the stratum with mixed stress levels between the parents in the couple strong evidence against MH was found for items 3 and 9. The results thus suggest that mothers are systematically more inclined to experience these symptoms than are their partner irrespective of the overall stress level in the couple. For item 3 the strongest tendency for higher mother agreement compared to the father in the couple was found in the stratum where both parents had high overall stress level (γ = 0.42, p<0.001). This was somewhat weaker in the stratum where only one parent had high overall stress scores (γ = 0.31, p = 0.003), and weakest in the stratum where both parents had low overall stress scores (γ = 0.24, p = 0.027). With regard to worrying about whether they do enough for their children (item 4), the strongest tendency was likewise found in the stratum with both parents with high overall stress levels (γ = 0.39, p = 0.003). When both parents in the couples have a high overall stress level, mothers are also systematically more inclined to agree that the child’s behavior often is embarrassing or stressful (item 13) than the fathers are (γ = 0.38, p<0.001). Regarding item 10 (*Having child(ren) leaves little time and flexibility in my life)*, evidence against MH was only found in couples with a low overall stress level (γ = 0.25, p = 0.01).

## Discussion

The aim of this study was to explore whether mothers and fathers in couples are equally inclined to experience a variety of parental stress symptoms measured by the PS subscale of the PSS. In a sample of parent couples with a child with physical and/or mental problems, we tested whether the experience of specific parental stress symptoms within the couple were in concordance with one another, when considering child gender, child age, parent couple educational level, and the overall parent couple stress level. In seven out of nine items, we found differences in agreement within the couples in relation to at least one of the stratification variables. We found systematic differences in agreement for five items. Only two parental stress symptoms presented agreement (i.e. MH) irrespective of child gender, child age, parent education or parental stress.

### Concordance and discordance within the couple

When we found systematic difference within the couples’ responses on an item, mothers were in all cases more inclined to report a higher degree of parental stress compared to the fathers. This is in agreement with previous studies examining differences between parental stress in mothers and fathers of children with developmental disabilities [13, 23, 25, 26, 34, 41–47]. The previous studies focus on the overall level of parental stress and compare mothers and fathers. Our study examines individual parental stress symptoms (item level), and takes the dependence in couple data into account, and thereby provides new insights into individual symptoms and the interdependence of mothers and fathers in couples.

It is likely that the dyadic coping strategies employed within the couple influence both the overall level of parental stress in a parent couple and the degree of concordance. Research has found beneficial effects if parents apply positive dyadic coping strategies [63, 65]. Benefits that apply both to the individual (e.g. better at handling stress, higher life satisfaction and lower levels of depression and anxiety), the couple (e.g. relationship satisfaction and stability), and children in the family (e.g. more prosocial behaviors and less behavior problems in children) [63, 65]. These issues are all relevant for the nine items in the PS subscale of the PSS. Generally, women more frequently engage in positive forms of dyadic coping, whereas men tend to engage in more negative dyadic coping [63]. This may contribute to understanding why mothers tend to experience higher levels of parental stress as they are not met with as much positive coping strategies as fathers are. Studies also find that parents with children with psychosocial challenges use more negative forms of dyadic coping [63].

We did not, however, find systematic differences in the mothers’ and fathers’ responses in all strata. Furthermore, there were no systematic differences between mothers and fathers in approximately half of the PS items. The items where we do not find any systematic differences (items 12, 14, 15, and 16), all tap into more general experiences of parental stress related to flexibility and control. It is possible that it is easier for parents to apply positive dyadic coping strategies on the issues concerned with having children that are at a more general level compared to the items that are on a more personal and daily level. For two of these items (14 and 16) mothers and fathers in the couples are equally inclined to agree irrespective child gender, child age, parent couple education, and overall stress level. For item 14 where parents were asked if they might not have children if they had to do it over again, more than 80% of both mothers and fathers either strongly disagree or disagree. This suggests that this item represents an extreme aspect of parental stress. Despite the challenges their child is experiencing, very few parents agree that they might not have children if they had to do it over again. Mothers and fathers are also equally inclined to agree on item 16 where they are asked if having children had meant too few choices and too little control over their life. Although item 16 does overlap with item 10 *Having child(ren) leaves little time and flexibility in my life* where we find a few differences, it seems as if parents agree more about issues around flexibility when they are asked at an overall level than at a more specific level.

We found systematic differences within the parent couples particularly in relation to three items: *Caring for my child(ren) sometimes takes more time and energy than I have to give* (item 3), *I sometimes worry whether I am doing enough for my child(ren)* (item 4): and *The major source of stress in my life is my children* (item 9). For these items, we find that mothers of boys, mothers with younger children, and mothers in couples where both had an educational level above high school were more inclined to agree compared to the father in the couples. Mothers were similarly generally more inclined to agree on these items irrespective of parent couple overall stress level. Common for items 3, 4, and 9 is that all three items tap into the specific personal experience of daily stressors related to having children and issues around feeling inadequate as a parent. This is also the case for items 10 where we see systematic differences in stress level between parent couples with a low overall stress level and for item 13 where mothers of boys are more inclined to agree that they often feel embarrassed or stressed by the child’s behavior than are the fathers in the couples.

One possible explanation for mothers being more inclined to report higher levels of these particular stress symptoms than fathers do in couples where both have a relatively long education may be related to the fact that in most Danish families both parents work [78]. When both parents work full time, there is still a tendency for mothers to be primarily responsible for the children [20] and working mothers with a longer education tend to have jobs with higher levels of responsibility. These mothers may therefore experience more daily stressors compared to the father in the couples.

Although not all studies find differences in parental stress related to child age [17, 18], some studies find that parents report lower levels of overall parental stress when the children get older [53–55] suggesting that parental stress is not stable across developmental periods [3]. Mothers of younger children tend to spend more time with their children around issues that can be stressful such as picking up from daycare/school, handling tired children, preparing dinner, and putting the children to bed [20, 60, 61]. This may also cause mothers of younger children to feel more stressed with regard to daily stressors (such as those addressed in item 3, 4, and 9) compared to the fathers. Furthermore, adolescents become more self-reliable and the parent-child interaction changes to a lower frequency in interactions (including affective exchanges) and more intense conflicts when the children become teenagers [56]. Thus, it is possible that parents of older children experience these specific stress symptoms to a similar degree because the challenges experienced with adolescents are of another character (such as issues with peers, screen time, and alcohol) and because behavior problems tend to decrease over time [18, 53].

An interesting finding with regard to the daily stressors is, that even in the group where both parents reported high overall parental stress (i.e. above the median cut-off-score), mothers were still more inclined to agree with the stress symptoms in items 3, 4, 9, and 13 compared to fathers in the couples. Previous research finds that parent couples characterized by conflict have more negative dyadic coping strategies, which lead to a lower well-being [63] and that fathers in these couples tend to withdraw more from the children than mothers [3]. This may contribute to mothers reporting more daily stressors than fathers.

### Parental stress and developmental disabilities

The degree to which parents experience specific parental stress symptoms may vary according to both the kind of challenges their child experiences and the level of symptoms. Previous research has found that parents of children with developmental problems (and particularly behavior problems) experience elevated levels of parental stress [10, 13–18, 25]. We were not able to conduct analyses conditioned on the kind of challenges that the child experiences, as we did not have exact information on diagnoses and symptom levels. However, we know that more than half of the sample comes from studies aimed at families with children with symptoms of ADHD or behavioral problems, which are more common in boys than in girls, and that the sample comprises around 75% boys. If the mothers tend to spend more time with their boys during the day than the fathers in the couples do, it is likely, that they experience a higher level of daily stressors than fathers [3], this might explain that mothers were more inclined to agree on items 3, 4, and 9. It is also possible, of course, that the explanation is that the mothers find the behaviors more problematic than the fathers do. This could also explain why mothers of boys tend to feel more embarrassed by their child’s behavior than the fathers do (item 13). It is also possible that mothers of children with developmental disabilities such as ASD and ADHD feel more embarrassed by their child’s behavior because they experience a higher degree of judgment from others than fathers do. Challenging child behaviors such as externalizing behavior and negative social attitudes are often perceived as abnormal and strange behavior by others and can easily be seen as a result of poor upbringing and poor parenting skills [19, 79, 80].

Parents of children with developmental disabilities may feel more isolated as a family and worry about their child’s future [19]. It is also common that they express a lack of support from professionals [19, 80]. As these families often experience high levels of parental stress which may lead to reduced parental responsiveness and child development [21] it is imperative to offer them appropriate interventions. Depending on how much the father participates in the parenting role there may be differences in the impact of intervention on mothers and fathers. One study finds that parenting stress in mothers of disabled children decreases when fathers parenting participation increases [81] and a recent meta-analysis examining the effects of interventions for parents of children with ADHD found larger effects on parental stress for mothers than for fathers [23].

### The strength of the statistical evidence

In a study of this nature where multiple tests are conducted, it is crucial to assess the effect of such multiple testing on the evidence. The effect of controlling the false detection rate (FDR) with the Benjamini-Hochberg procedure was mainly that the strength of the evidence was reduced from being strong to being moderate in some cases, but results remained significant. Only the results for item 3 in the strata where only one parent had education above high school level and where both parents had low overall stress scores, respectively, became insignificant when controlling the FDR. However, as the evidence against MH was already very weak in these two instances, we do not consider this a relevant change in the results.

It is obvious that for some of the smaller strata, insignificant results (i.e. evidence of marginal homogeneity) might be due to a lack of power. Consideration of the directionality of the results across the single strata in a covariate is thus important, as result in the same direction across strata solidifies the findings and they might be described by the partial results. Thus, when considering child gender, the tendency for mothers to be more prone than fathers to agree with the particular item statement of item 3, 4 and 9 than the fathers are is accentuated for parents of boys, but is also present for parent of girls, and the partial correlations considering child gender are relatively strong. Turning to stratification by child age there appears to be a pattern, where the tendency for mothers to rate items 3, 4 and 9 higher than fathers is not very different for the two youngest age groups of the children, but showing a declining trend as the children are older never the less. Considering the educational level of the parent couples, it is noticeable that the tendency for mothers to agree more with the items 3, 4 and 9 is the strongest when both parents have an educational level above high school. Lastly, when considering the overall stress level of the parent couples, the directionality of the results differ across items 3, 4 and 9: for item 3, the tendency for mother to agree more than fathers is accentuated with the overall stress level in the couple, for item 9 the opposite pattern can be seen, and for item 4 there is not a clear directionality pattern.

### Strengths, limitations and future studies

The present study has a number of strengths which contributes to the field of research in entirely new ways. Firstly, this study is the first to explore differences in the single parental stress symptom level for mothers and fathers, as opposed to the score level, which is the norm in previous studies. Secondly, the study is the first to address these differences with a within-couple design, thus taking the relatedness of mothers and fathers into account. Third, and finally, we studied parental stress items from an instrument which has been successfully validated using state-of-the-art item response theory methods in the Danish context, including parents of children with behavior problems [34].

This study also has some limitations. Firstly, the nature of the problems a child experiences may affect the experience of specific aspects of parental stress. As we did not have access to the specific diagnoses and severity of disease/problems in this study, we could not include such stratification. This, we consider a crucial study to undertake in the future. Secondly, the covariates used in our study may interact with each other. It was, however, not possible to test for interactions. Additionally, the relatively small sample size did not allow for stratification of the analyses by more than one covariate at a time. Thus, future studies with larger samples of parent couples are warranted. Thirdly, few couples in the sample had a relatively low educational level (high school or less). Therefore, it was not possible to conduct analyses among this group of parents. Previous research finds that mothers with short educations tend to show higher levels of parental stress than mothers with a medium or long education do [30, 57–59]. Parents with short education are also at higher risk of experiencing other challenges that also influence the level of parental stress such as poverty, mental health problems, adverse childhood experiences, and lack of social support [2–7]. These factors may all influence how mothers and fathers experience specific symptoms of parental stress. Future studies should therefore examine parental concordance in a more disadvantaged sample. Finally, many of the families included in the study are likely to have more than one child, but we could not access information on additional children. Being a systemic construct, parental stress is likely cumulative and influenced by the prevalent coping strategies within the couples and e.g. the number and age of the children currently living in the family [3]. Thus, we finally suggest that future studies take into account the number of children in the families and their ages, and assessment of dyadic coping strategies. More encompassing studies could provide insight as to how differences in the experience of specific aspects of parental stress between mothers and fathers are associated with the couples coping strategies.

## Conclusion

This study suggests that in parent couples of children with physical and/or mental health issues, the mothers and fathers’ experience of some specific aspects of parental stress vary, while others do not. Furthermore, these differences may also be influenced by child and couple characteristics. Mothers of boys, mothers of younger children, and mothers in couples with an education above high school were more inclined to agree on items related to daily stressors compared to the father in the couple. These findings may help inform future interventions (e.g. parenting interventions) by focusing especially on these subgroups of mothers and whether fathers can be involved as a supportive resource. Future studies should therefore take into account the systemic nature of parental stress and include more aspects of family life in vulnerable families. This includes examining concordance and discordance in perception of specific aspects of parental stress depending on child diagnosis, number of children, and coping strategies within the couple.

## Supporting information

S1 Table Test for marginal homogeneity: Crude results. (DOCX)

S2 Table Marginal frequencies for item 10 in the child gender stratum, parents of girls. (DOCX)

S3 Table Marginal frequencies for item 4 in the couple educational level stratum, where one parent has an educational above high school. (DOCX)
