# Supplementary material for: Concordance within parent couples’ perception of parental stress symptoms among parents to 1-18-year-olds with physical or mental health problems
Source: PLoS One. 2020 Dec 18;15(12):e0244212. doi: 10.1371/journal.pone.0244212 (PMC7748276; doi:10.1371/journal.pone.0244212)
Supplement: S1 Table — (DOCX) [file pone.0244212.s001.docx]

**S1 Table. Test for marginal homogeneity: Crude results**

|  |  | **G^2-^test** | | |  | **γ-test** | |
| --- | --- | --- | --- | --- | --- | --- | --- |
| Item |  | G^2^ | df | p |  | γ _m-f_ | p |
| 3 |  | 30.4 | 4 | <.001 |  | .30 | <.001 |
| 4 |  | 31.2 | 4 | <.001 |  | .30 | <.001 |
| 9 |  | 26.6 | 4 | <.001 |  | .26 | <.001 |
| 10 |  | 4.9 | 4 | .298 |  | .11 | .023 |
| 12 |  | 2.4 | 4 | .670 |  | -.03 | .290 |
| 13 |  | 19.2 | 4 | .001 |  | .17 | .001 |
| 14 |  | 1.6 | 4 | .814 |  | -.06 | .219 |
| 15 |  | 3.2 | 4 | .527 |  | .05 | .232 |
| 16 |  | 1.8 | 4 | .769 |  | -.0.8 | .108 |

^*^ The Benjamini-Hochberg (1995) procedure rejects tests with p-values less than 0.0250 to fix the false discovery rate (FDR) at 5 % and at 0.0044 for FDR = 1 %.
