# Supplementary material for: Concordance within parent couples’ perception of parental stress symptoms among parents to 1-18-year-olds with physical or mental health problems
Source: PLoS One. 2020 Dec 18;15(12):e0244212. doi: 10.1371/journal.pone.0244212 (PMC7748276; doi:10.1371/journal.pone.0244212)
Supplement: S2 Table — (DOCX) [file pone.0244212.s002.docx]

**S2 Table.** Marginal frequencies for item 10 in the child gender stratum, parents of girls.

|  | **Strongly disagree** | | **Disagree** | | **Undecided** | | **Agree** | | **Strongly agree** | | **Total** | |
| --- | --- | --- | --- | --- | --- | --- | --- | --- | --- | --- | --- | --- |
|  | n | % | n | % | n | % | n | % | n | % | n | % |
| Fathers | 1 | 1.9 | 11 | 21.2 | 11 | 21.2 | 20 | 38.5 | 9 | 17.3 | 52 | 100 |
| Mothers | 4 | 7.7 | 6 | 11.5 | 11 | 21.2 | 17 | 32.7 | 14 | 26.9 | 52 | 100 |
| Total | 5 | 4.8 | 17 | 16.3 | 22 | 21.2 | 37 | 35.6 | 23 | 22.1 | 104 | 100 |
