# Supplementary material for: Concordance within parent couples’ perception of parental stress symptoms among parents to 1-18-year-olds with physical or mental health problems
Source: PLoS One. 2020 Dec 18;15(12):e0244212. doi: 10.1371/journal.pone.0244212 (PMC7748276; doi:10.1371/journal.pone.0244212)
Supplement: S3 Table — (DOCX) [file pone.0244212.s003.docx]

**S3 Table.** Marginal frequencies for item 4 in the couple educational level stratum, where one parent has an educational above high school

|  | **Strongly disagree** | | **Disagree** | | **Undecided** | | **Agree** | | **Strongly agree** | | **Total** | |
| --- | --- | --- | --- | --- | --- | --- | --- | --- | --- | --- | --- | --- |
|  | n | % | n | % | n | % | n | % | n | % | n | % |
| Fathers | 3 | 2.3 | 11 | 8.3 | 22 | 16.5 | 65 | 48.9 | 32 | 24.1 | 133 | 100 |
| Mothers | 6 | 4,5 | 8 | 6.0 | 5 | 3.8 | 54 | 40.6 | 60 | 45.1 | 133 | 100 |
| Total | 9 | 3.4 | 19 | 7.1 | 27 | 10.2 | 119 | 44.7 | 93 | 34.6 | 266 | 100 |
